# Triple drug therapy with GABA, sitagliptin, and omeprazole prevents type 1 diabetes onset and promotes its reversal in non-obese diabetic mice

**DOI:** 10.3389/fendo.2022.1028114

**Published:** 2022-10-21

**Authors:** Francisco Alejandro Lagunas-Rangel, Daniil Koshelev, Andrej Nedorubov, Liudmila Kosheleva, Vladimir Trukhan, Alexander Rabinovitch, Helgi B. Schiöth, Shmuel Levit

**Affiliations:** ^1^ Department of Surgical Sciences, Functional Pharmacology and Neuroscience, Uppsala University, Uppsala, Sweden; ^2^ Levicure LTD, Tel Aviv, Israel; ^3^ Advanced Molecular Technology LLC, Moscow, Russia; ^4^ Kelowna, BC, Canada; ^5^ Institute of Endocrinology, Diabetes & Metabolism, Tel Aviv, Israel

**Keywords:** type 1 diabetes (or diabetes), NOD mice, insulin, GABA, DPP-4 inhibitor, PPI

## Abstract

Previous studies have reported that dual drug combinations consisting of γ-aminobutyric acid (GABA) together with a dipeptidyl-peptidase-4 inhibitor (DPP-4i), also a DPP-4i with a proton pump inhibitor (PPI), could improve pancreatic β-cell function and ameliorate diabetes in diabetic mice. In this study, we sought to determine if a triple drug combination of GABA, a DPP-4i and a PPI might have superior therapeutic effects compared with double drug therapies in the prevention and reversal of diabetes in the non-obese diabetic (NOD) mouse model of human type 1 diabetes (T1D). In a diabetes prevention arm of the study, the triple drug combination of GABA, a DPP-4i, and a PPI exhibited superior therapeutic effects in preventing the onset of diabetes compared with all the double drug combinations and placebo. Also, the triple drug combination significantly increased circulating C-peptide and serum insulin levels in the mice. In a diabetes reversal arm of the study, the triple drug combination was superior to all of the double drug combinations in reducing hyperglycemia in the mice. In addition, the triple drug combination was the most effective in increasing circulating levels of C-peptide and serum insulin, thereby significantly reducing exogenous insulin needs. The combination of GABA, a DPP-4i and a PPI appears to be a promising and easily scalable therapy for the treatment and prevention of T1D.

## Introduction

Type 1 diabetes (T1D) is a disease characterized by a lack of insulin production by pancreatic islet β-cells due to their autoimmune destruction (1, 2). In the United States, the overall pediatric incidence of T1D is approximately 25 per 100,000 per year and increased by 2% to 3% per year during the period 2002-2012 (3). T1D develops mainly in childhood or adolescence and, to date, there is no known way to reverse or prevent it (4). Treatment options are very limited and most T1D patients rely solely on lifelong insulin therapy for survival (5). The high costs of new technologies based on advanced insulin treatments reduce their universal accessibility (6). Procedures such as islet or pancreas transplantation cannot be considered scalable and consistently reliable treatment options, due to a number of limiting factors including their extreme complexity, donor availability, requirement for life-long immunosuppression, and high cost (7). Indeed, only autologous stem cell-derived islet transplants are considered as a future therapeutic option (6). With early diagnosis of the disease, some β-cell functions persist for a short period of time, although this is insufficient to maintain normoglycemia without exogenous insulin injection (8). Even minimally preserved β-cell function can prevent the development of diabetic complications (9, 10). Therefore, any therapy that can offer protection and maintenance of residual β-cell function is of paramount importance.

Repurposing of approved medical compounds and finding synergistic drug combinations has been identified as a viable and easily scalable treatment option for complex diseases with no available effective medications, such as T1D (11).

Therapeutic agents for type 2 diabetes such as DPP-4i are considered safe and have been extensively researched for decades (12). DPP4-i were shown to inhibit approximately 80–90% of DPP-4 activity (13–15) leading to a 2–3-fold elevation in glucagon-like peptide-1 (GLP-1) (16),thereby prolonging the stimulation of GLP-1 receptors (GLP-1R) and activating key transcription factors for β-cell growth and survival (17). Furthermore, drugs in the DPP4-i family also reduce inflammation (insulitis), and enhance pancreatic β-cell regeneration (18). Addressing immunomodulation in T1D, DPP-4i have also been reported to effectively target autoimmunity through down-regulating Th1-like immune cells, up-regulating Th2-type cytokines, promoting Treg cell proliferation, and decreasing IL-17 production (19).

Effects of DPP-4i drugs in diabetic animal models showed some promise through improving islet neogenesis and β-cell survival (20–24). Selected clinical studies have reported on the therapeutic effects of DPP-4i in humans with T1D, specifically in the reduction of insulin requirements and inhibition of glucagon secretion (25, 26). However, these findings remain controversial and did not translate successfully in T1D human clinical trials, showing no significant improvement in blood glucose, C-peptide, or HbA1c levels (27, 28).

γ-aminobutyric acid (GABA) is an important neurotransmitter that was found to induce membrane hyperpolarization and suppress glucagon secretion by pancreatic islet α-cells whereas in β-cells it induced membrane depolarization and increased insulin production (29). Importantly, GABA has an overall anti-inflammatory effect on the immune system and was shown to suppresses lymphocyte proliferation through GABA-A receptors (30). Additionally, induction and activation of the GABA-B receptor in human islets modulates insulin secretion (31). GABA has also been reported to induce α-cell transdifferentiation into β-like cells through the GABA-A receptor (32).

The therapeutic efficacy of GABA was significantly improved with the addition of the DPP-4i, sitagliptin in murine pancreatic islets and the combination increased β-cell mass and proliferation as well as reduced β-cell apoptosis (33).

Another family of well-researched drugs, PPI, inhibit H+-K+-ATPase and increase the concentration of gastrin (a stimulant for pancreatic β-cell regeneration) in blood and pancreatic tissue (34). Combining a DPP-4i with a PPI was reported to enhance the synergistic effect of the two drugs, restore normoglycemia in NOD mice (35) and induce β-cell neogenesis from human pancreatic duct cells implanted in NOD-severe combined immunodeficient mice (36).

Here, we sought to determine if there might be an improved therapeutic effect of the oral administration of a three-drug combination consisting of sitagliptin (a DPP-4i), omeprazole (a PPI) and GABA as a novel pharmacological treatment of T1D. We compared the effects of the triple drug combination with those of the double combinations and placebo, both before and after diabetes onset in NOD mice.

## Material and methods

### Animal ethics

The experimental procedures were carried out in accordance with the Decision of the Council of the Eurasian Economic Commission No. 81 dated 03/11/2016 “On the approval of the rules of good laboratory practice of the Eurasian Economic Union in the field of circulation of medicines”; GOST R 33044-2014 “Principles of good laboratory practice”; US FDA Good Laboratory Practice (GLP) Regulations for Non-clinical Laboratory Studies (21 CFR Part 58); guidelines of the European Federation of Laboratory Animal Science Associations, based on European Union legislation (Directive 2010/63/EU), as well as the NIH Guide for the care and use of laboratory animals, local laws and policies. The procedures were approved by the ethics committee of the I. M. Sechenov First Moscow State Medical University (Approval Code PRC-043 with start date October 28, 2020).

### Animals

Female non-obese diabetic (NOD)/ShiLtJ mice (The Jackson Laboratories, IMSR_JAX:001976) weighing 18-20 grams and 10-11 weeks old were used in this study. At the time of initiation of therapy, the animals had reached the age of 17-18 weeks. The animals were kept in polycarbonate cages with sterilized non-coniferous softwood sawdust bedding under controlled conditions of temperature (23°С ± 3) and humidity (55% ± 15) and with a light-dark cycle of 12:12 hours. Water and standard chow (Safe, D04) were available *ad libitum* at all times.

### Group formation, drug preparation and administration

NOD mice were identified as diabetic by blood glucose (BG) monitoring starting at 10–11 weeks of age twice a week. Diabetes was defined as a random BG >13.9 mmol/L in two consecutive measurements on different days. The randomization was initiated after 30% of the cohort were diagnosed with diabetes. Thereafter, BG was measured on four consecutive days to reaffirm the diabetes diagnosis, and randomization in five groups was performed.

NOD mice between 17 and 18 weeks of age were equally divided into 5 groups: 1. Placebo, 2. GABA, sitagliptin, and omeprazole (designated A+B+C), 3. GABA and sitagliptin (designated A+B), 4. GABA and omeprazole (designated A+C), and 5. Sitagliptin and omeprazole (designated B+C). After that, two study arms were formed based on BG levels in the mice: a) a diabetes prevention arm that involved 94 NOD mice before the onset of diabetes and b) a diabetes reversal arm that involved 51 NOD mice with confirmed T1D status. For the treatments, GABA 250 mg (Aminalon) and sitagliptin 100 mg (Januvia) film-coated tablets, as well as omeprazole 20 mg (Omeprazole Teva) enteric capsules were used. The pharmaceutical forms were dissolved in a 1% starch solution and were delivered by oral gavage to the mice twice a day in a volume of 1 mL in the morning at 10:00-11:00 and in the afternoon at 18:00-19:00. The doses used were GABA 300 mg/kg, sitagliptin 30 mg/kg, and omeprazole 15 mg/kg. The placebo group received 1% starch solution. The drugs were administered for 70 days.

Along with the drugs, the severely diabetic mice were given insulin glargine (Lantus) according to the specific “Sliding scale” protocol shown in **Table 1**. The insulin therapy was only administered to mice in severe hyperglycaemia with blood glucose levels over 30 mmol/L. Mice with blood glucose lower than 30 mmol/L were not treated with exogenous insulin injections.

**Table 1:** Dosing and administration of insulin.

Blood glucose range	Dose of insulin glargine
30.0- 33.3 mM/L	1.5 units/day
> 33.3 mM/L in a single measurement	1.5 units - morning 1 unit - evening
> 33.3 mM/L in two consecutive measurements	1.5 units - morning 1.5 units in the evening

### Blood glucose determination

Non-fasting blood glucose levels in mice were measured three times per week for 70 days with an AccuCheck Performa glucometer (Roche) and AccuCheck Performa test strips (Roche) following the supplier’s instructions. Blood was taken from the tail vein.

### Weighing

The mice were weighed once per week for 70 days with an M-ER 122ACFJR-600.01 LCD balance (Mertech).

### Plasma collection

Approximately 700 µL of blood was sampled from the ophthalmic sinus of fasted mice (4 hours without food) using tubes with K+EDTA in the afternoon (12-2 PM) at weeks 2, 5 and 10. Plasma was obtained by centrifuging the blood for 10 minutes at 3500 rpm at 4°C. The plasma samples were frozen and kept at -20°C until the measurement of the different parameters.

### C-peptide determination

A 96-well plate (Corning, 3590) was incubated for 16-18 hours at 40°C with C-peptide polyclonal antibodies (Invitrogen, PA5-85595) diluted in 20 mM carbonate buffer (Panreac, 571648.201638) at a concentration of 200 ng/100 μL. Unbound immunoglobulins were removed by three serial washes with PBST (1X PBS and 0.01% Tween 20). To block non-specific binding sites, the plate was incubated during 30 minutes at 37°C with 200 µL of blocking buffer (BB) composed of 20 mM Tris-HCl buffer (Panreac, 141940.1211), 0.14 M NaCl (Panreac, 141659.1214) and 0.5% skimmed bovine milk powder (Sigma, M7409). Then, three serial washes with PBST were performed and 100 μL of BB containing increasing concentrations of C-peptide (0, 3.9, 7.8, 15.6, 31.25, 62.5, 125, 250 ng/mL) were added to 12 wells to create a curve. In parallel, 100 µL of test plasma diluted 3000-fold with BB was added to the other wells. Plates were incubated for 30 minutes at 37°C. The contents of the wells were removed and washed 3 times with 200 µL of 20 mM Tris-HCl buffer containing 0.05% Tween-20 (Panreac, 162312.1611). 100 µL of a BB containing biotin-labeled rabbit polyclonal anti-C-peptide antibody (Sigma, H1759) at a concentration of 200 ng/100 µL was added to the wells and incubated for 30 minutes at 37°C. After incubation, the contents of the wells were aspirated and peroxidase-conjugated avidin (Sigma, A3151) was added and incubated for 30 minutes at 37°C. The contents of the wells were aspirated, the wells were washed 4 times with 200 µL of 20 mM Tris-HCl containing 0.05% Tween 20 and subsequently 100 µL of 0.1 M sodium acetate buffer (Panreac, 141008.1611), 0.1 mg/mL tetramethylbenzidine (Sigma, T8768), and 0.003% H_2_O_2_ (Sigma-Aldrich, H1009) were added to the wells. To stop the reaction, 50 µL of 2M H_2_SO_4_ (chemically pure, KhimMed LLC) was added to the wells and the optical density at 450 nm was measured. The concentration of C-peptide in the samples was determined based on the curve created.

### Insulin determination

Serum insulin determination was performed with a protocol similar to that of C-peptide, except that in this case polyclonal insulin antibodies (Bioss, BS-0056R) were used.

### Glucagon determination

Serum glucagon levels were measured with the mouse GCG/glucagon ELISA kit (LSBio, F5904) according to the supplier’s instructions.

### Statistical analysis

Data processing was performed in GraphPad Prism v. 9.1 (GraphPad Software, San Diego, California USA) and using Python with the libraries: NumPy, Scipy, Statsmodels, Lmfit, Lifelines, Pandas, Matplotlib, Seaborn, Scikit-postthoc. The Kruskal-Wallis test with Dunn’s *post hoc* was used to compare three or more groups. The false discovery rate method, the two-stage Benjamini-Hochberg procedure, was used as the method to control the level of significance. To represent the change in glucose concentration and mass, the median was used as a measure of central tendency and the interquartile width as a measure of dispersion. To visualize between-group differences for other data, “whisker boxes” representing the median, interquartile width, and range were used. To describe the cumulative insulin consumption curve, an approximation to the sigmoid shape was applied:


$$y=\frac{C_{1}}{\left(1+e^{-C_{2}x-C_{3}}\right)}$$

optimize the parameters of the curve, the Levenberg-Marquardt algorithm was used using the initial values ​​C1 = 500, C2 = 0.02, C3 = 25. Model quality was assessed using probability plots and Cramer-von Mises and Kolmogorov-Smirnov goodness-of-fit tests. According to the criteria, the model adequately describes the data under study. Using the sigmoid model, the days on which there was a decrease in the rate of insulin consumption were determined. The significance level of 5% (α = 0.05) was chosen as the threshold to reject the null hypothesis in all the tests used.

## Results

### Combined use of GABA, sitagliptin, and omeprazole prevents the onset of diabetes in NOD mice

To assess the potential additive therapeutic effects of the triple combination of GABA, sitagliptin, and omeprazole (A+B+C) in preventing the onset of diabetes in NOD mice, mice 17 -18 weeks of age were administered placebo, double, and triple combination therapies for 10 weeks.

**Figure 1:**
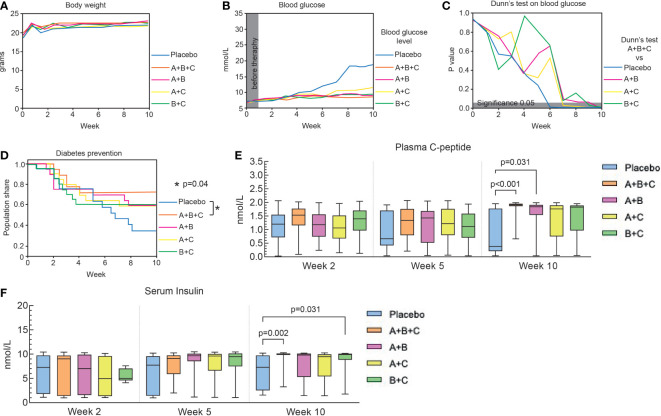
Triple therapy (A+B+C) prevents diabetes onset in NOD mice. This arm of the experiments was performed on 94 mice that had not developed diabetes at the start of drug therapy (blood glucose<13.9. mmol/L). The placebo, A+B+C, A+B and A+C groups included 19 mice each and the B+C group 18. **(A)** Changes in body weight of the mice in each group are shown as median values. The data were analyzed with the Kruskal-Wallis test with Dunn’s *post hoc*. No significant changes were found between the groups. **(B)** Changes in median blood glucose levels in each group over 10 weeks. **(C)** Blood glucose levels of the A+B+C group were compared with the other groups at different times using the Kruskal-Wallis test with Dunn’s *post hoc*. The gray area corresponds to a level of significance (p = 0.05). **(D)** Kaplan-Meier curves of the proportion of mice that were prevented from diabetes development. There was a significant difference between the A+B+C group and the placebo group (p = 0.04). No statistical differences were found when comparing the placebo group with the dual therapy groups (A+B [p=0.22], A+C [p=0.23], B+C [p=0.22]). **(E)** Plasma C-peptide levels were analyzed with the Kruskal-Wallis test with Dunn’s *post hoc*. Significant differences were found by comparing the placebo group with A+B+C (p<0.001) and with A+B (p= 0.031) groups at week 10. **(F)** Serum insulin levels were analyzed with the Kruskal-Wallis test with Dunn’s *post hoc*. Significant differences were found by comparing the placebo group with the A+B+C (p=0.002) and with the B+C (p=0.031) groups at week 10. Plasma C-peptide and serum insulin levels are shown as box-whisker plots representing median values with interquartile width and range.

Mice in all groups gradually gained weight with age, and there were no significant differences between the groups (**Figure 1A**).

At randomization (week 0), median blood glucose levels were similar (6.4 – 6.7 mmol/L) among the groups (**Figure 1B**). By week 10, the median blood glucose level for group A+B+C (8.2 mmol/L) was significantly lower than that in the placebo group (18.7 mmol/L, p<0.001) and all double combinations. Also, blood glucose levels in groups A+B (8.9 mmol/L, p<0.001), A+C (10.6 mmol/L, p<0.001), and B+C (9.2 mmol/L, p<0.001) were significantly lower than in the placebo group (**Figure 1C**).

**Figure 1D** shows that 12 out 18 (67%) mice in the placebo group had developed diabetes (blood glucose >13.9 mmol/L in two repeated measurements) by week 10, whereas only 5 out of 18 (28%) of mice in the A+B+C group were diabetic at that time (p=0.04, A+B+C *vs.* placebo). Diabetes also developed in 8 out of 19 (42%) mice in A+B and A+C groups and in 8 out of 20 (40%) mice in B+C group, but this was not significantly different from the 67% diabetes development in the placebo group at 10 weeks.

To monitor the effects of the treatments on pancreatic β-cell function, plasma C-peptide and serum insulin levels were measured at weeks 2, 5 and 10. Plasma C-peptide levels in the placebo group dropped from 1.13 nmol/L at 2 weeks to 0.38 nmol/L at 10 weeks, whereas all other groups showed elevation of the median C-peptide level. However, only A+B+C (p<0.001) and A+B (p=0.031) combination therapies showed significant increases in C-peptide levels compared with the placebo group at week 10 (**Figure 1E**). Serum insulin levels were also increased significantly in A+B+C (p = 0.002) and B+C (p = 0.031) groups compared with the placebo group (**Figure 1F**). Notably, A+B+C was the only group to show significant increases in both C-peptide and insulin levels compared with the placebo group.

### Combined use of GABA, sitagliptin, and omeprazole promotes diabetes reversal in NOD mice

To evaluate the potential additive therapeutic effects of the triple combination of GABA, sitagliptin, and omeprazole (A+B+C) in treating NOD mice 17-18 weeks of age with confirmed diabetes (blood glucose >13.9 mmol/L), the mice were administered placebo, double, and triple combination therapies for 10 weeks.

**Figure 2:**
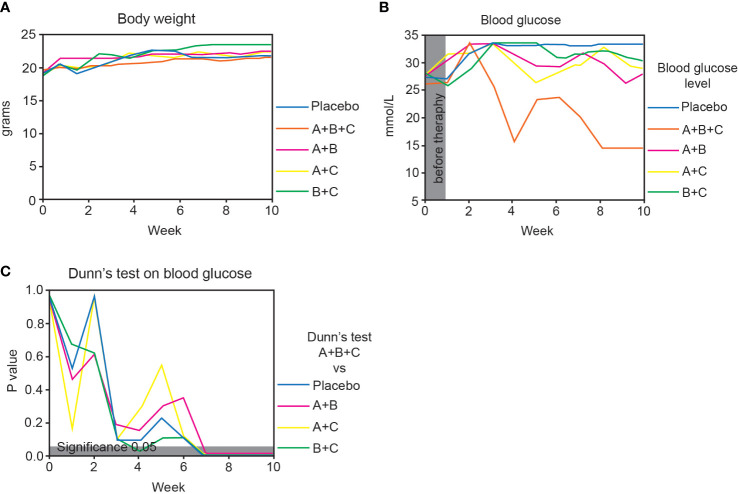
Triple therapy (A+B+C) promotes diabetes reversal in NOD mice. This arm of the experiments was performed on 51 mice that had developed diabetes at the start of drug therapy (blood glucose >13.9 mmol/L). The placebo, A+B, A+C and B+C groups included 10 mice each and the A+B+C group 11. **(A)** Changes in body weight of the mice in each group are shown as median values. The data were analyzed with the Kruskal-Wallis test with Dunn’s *post hoc*. No significant changes were found between the groups. **(B)** Changes in median blood glucose levels in each group over 10 weeks. **(C)** Blood glucose levels of the A+B+C group were compared with the other groups at different times using the Kruskal-Wallis test with Dunn’s *post hoc*. The gray area corresponds to a level of significance (p = 0.05).

There were no significant differences in weight changes with aging in the different groups (**Figure 2A**).

At randomization (week 0), median blood glucose levels were similar (26.0 – 28.0 mmol/L) among the groups (**Figure 2B**). At week 10 there was a highly significant difference in the median blood glucose level in the A+B+C group (14.5 mmol/L) compared with the placebo group (33.3 mmol/L, p=0.001). Also, blood glucose in the A+B+C group (14.5 mmol/L) was significantly lower than that in all double combination groups: A+B (28.1 mmol/L, p<0.001), A+C (28.6 mmol/L, p<0.001), and B+C (30.2 mmol/L, p<0.001) (**Figure 2C**).

### Combined use of GABA, sitagliptin, and omeprazole decreases insulin requirements and increases C-peptide and insulin levels in diabetic NOD mice

Insulin therapy was used to manage severely hyperglycemic mice (blood glucose > 30 mmol/L) in all treatment groups, and the total insulin intake was measured to determine the efficacy of the treatments in reducing insulin requirements.

By week 10 in the diabetes reversal arm of the experiments, the triple therapy (A+B+C) group had fewer mice dependent on insulin therapy compared with all other groups. The A+B+C group had the largest proportion of mice that discontinued insulin treatment: 9 of 10 mice (90%) compared with 7 of 9 mice (78%) in group A+B, 6 of 9 mice (67%) in group A+C, 3 of 9 mice (33%) in group B+C, and only 1 of 8 mice (13%) in the placebo group.

It is important to additionally point out that in the reversal arm of the study only mice with BG levels over 30 mmol/L were treated with exogenous insulin injections in accordance with “Sliding scale” protocol (**Table 1**). 5 mice in the reversal arm of the study did not reach BG levels higher than 30 mmol/L and, therefore, were not treated with exogenous insulin injections during the experiment.

**Figure 3:**
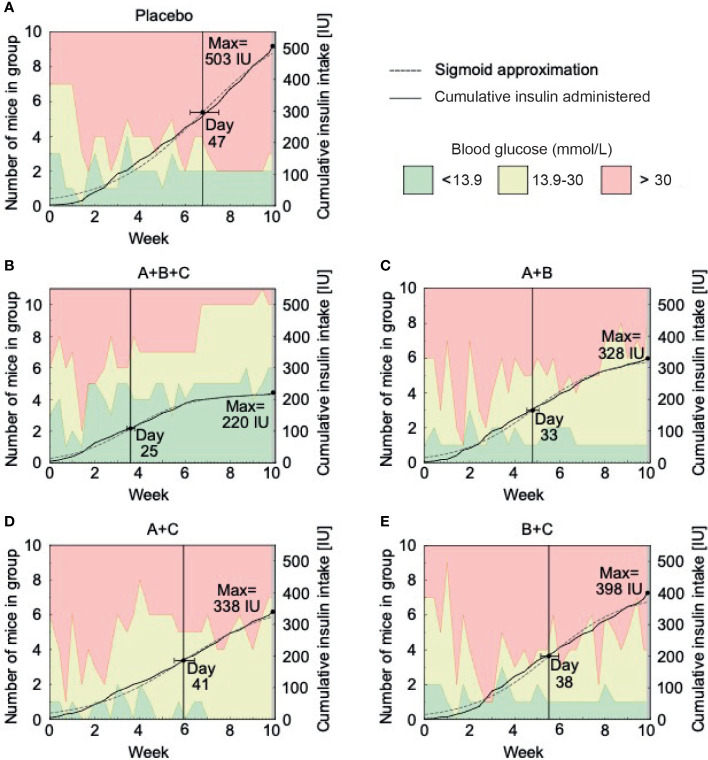
Insulin requirements decrease with triple therapy (A+B+C) in NOD mice. This arm of the experiment was performed on 51 mice with confirmed diabetes (blood glucose >13.9 mmol/L) at the start of drug therapy. The placebo **(A)**, A+B **(C)**, A+C **(D)** and B+C **(E)** groups included 10 mice each and the A+B+C **(B)** group included 11 mice. The graphs show the number of mice within the different glucose levels (green area represents normoglycemic mice with blood glucose levels below 13.9 mmol/l, yellow represents hyperglycemic mice without insulin therapy with blood glucose levels between 13.9 - 30.0 mmol/l and red represents severely hyperglycemic mice on insulin therapy with blood glucose levels above 30.0 mmol/l) and the cumulative amount of insulin administered to them during each week of the study. Additionally, a sigmoid approximation model of the exogenous insulin used in each group and the anticipated day of a reduced insulin administration intensity are displayed.

On **Figure 3** sigmoid approximation of cumulative insulin intake shows that the A+B+C group’s decrease in exogenous insulin demands occurred earlier (day 25) than in the placebo group (day 47) and the dual therapy groups: A+B (day 33), A+C (day 41), and B+C (day 38). As expected, the placebo group had the highest insulin demands.

**Figure 4:**
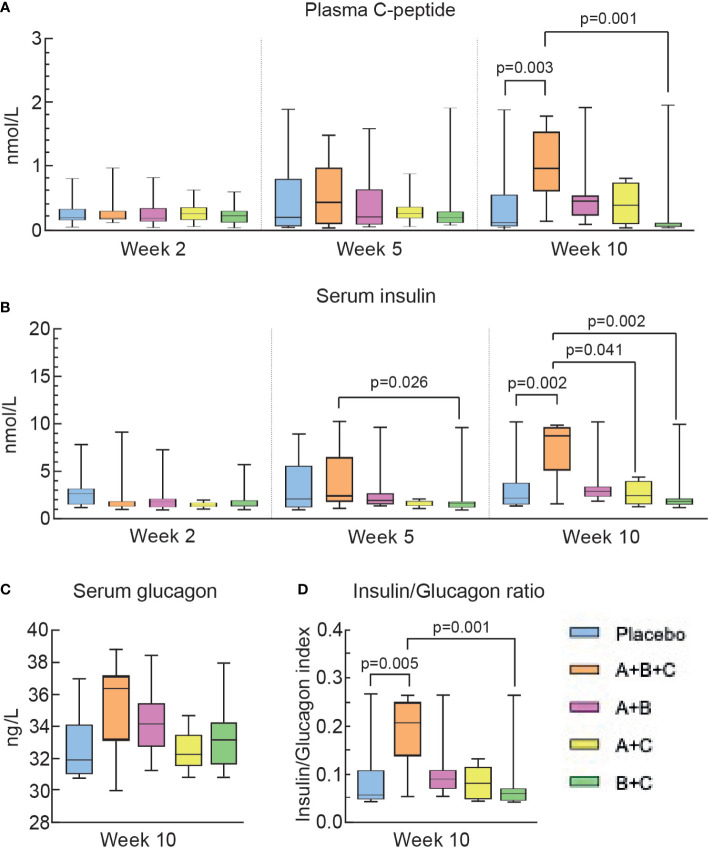
Triple therapy (A+B+C) increases C-peptide and insulin levels in diabetic NOD mice. Differences in the levels of plasma C-peptide **(A)**, serum insulin **(B)**, serum glucagon **(C)**, and the insulin/glucagon ratio **(D)** were analyzed using the Kruskal-Wallis test with Dunn’s *post hoc*. The box-whisker plots represent median values with interquartile width and range.

Plasma C-peptide and serum insulin levels were measured at weeks 2, 5 and 10 to monitor the effects of the combination treatments on pancreatic β-cell function. Soon after the beginning of treatments (week 2), median C-peptide levels were similar (0.17-0.25 nmol/L) among the groups (**Figure 4A**). By week 10, the C-peptide level in group A+B+C increased more than 5-fold to 0.93 nmol/L and was significantly higher than in the placebo group (0.06 nmol/l, p=0.003) and the B+C group (0.07 nmol/l, p=0.002), both of which saw decreases in C-peptide levels from weeks 2 to 10. C-peptide levels increased slightly from 2 to 10 weeks in groups A+B (0.43 nmol/l, p=0.216) and A+C (0.38 nmol/l, p=0.521), but did not reach significance compared with the placebo group (**Figure 4A** **).**

Serum insulin levels were similar (1.29-1.76 µU/ml) among the groups at week 2 (**Figure 4B**). By week 10,insulin levels in groups A+B+C increased more than 6-fold to 8.75µU/ml and this was significantly higher than in the placebo group (1.65 µU/ml, p=0.003), the B+C group (1.79 µU/ml, p=0.002) and the A+C group (2.68 µU/ml, p=0.041, but did not reach significance compared with group A+B (2.92 µU/ml, p=0.49). The A+B+C group was the only group to show a significant increase in serum insulin compared with the placebo group (**Figure 4B**), which is compatible with this A+B+C group having required significantly less exogeneous insulin therapy than all other groups (**Figure 3**).

Serum glucagon levels were measured once in week 10. (**Figure 4C** **).** Serum glucagon tended to increase in the A+B+C group and the insulin/glucagon ratio (**Figure 4D**) was significantly higher in mice in the A+B+C group than in the placebo group (p=0.005).

## Discussion

In this study, we found that a triple drug combination therapy consisting of GABA, a DPP-4i and a PPI (designated A+B+C) has a superior therapeutic effect in improving diabetes parameters in NOD mice, an animal model for human T1D. The A+B+C triple drug combination was superior to all the double drug combinations in preventing diabetes onset, and this was accompanied by significant increases in circulating levels of C-peptide and insulin. In addition, the triple drug combination was most effective at lowering blood glucose levels in severely diabetic mice and was the only drug combination to significantly increase C-peptide and insulin levels in the mice, together with decreased requirements for exogenous insulin therapy.

To determine the appropriate duration of the experiment, various parameters, including renewal of heterogeneous cells in the mouse pancreas (37) and previous studies with GABA, DPP-4i, and PPI (32, 33, 35) were taken into account to demonstrate the efficacy of the long-term therapy administration in mouse T1D models. It was concluded that in order for the study to yield conclusive evidence on the efficacy of the combination treatment in NOD mice, the triple combination therapy should be administered for 70 days or 10 weeks.

In this study, the NOD mouse with spontaneous autoimmune diabetes (38, 39) was deliberately chosen to assess the potential of additive therapeutic effect of drug combinations in T1D. A major strength of the NOD model for human T1D compared to diabetes induced by chemical substances (STZ, alloxan, dithizone) (38, 40, 41) is the similarity to human T1D, as the NOD mice develop pancreatic islet β-cell autoantibodies (42) and their islets are infiltrated with CD4+ and CD8+ lymphocytes (40).

Insulin replacement therapy is required for all individuals with T1D and consists of multiple daily injections (MDI) of basal and prandial insulin or a continuous subcutaneous insulin infusion (43). To determine whether any of the combination treatments could decrease or completely remove the requirement for insulin therapy, our NOD model study was designed to resemble T1D disease management in humans. In our study, exogenous insulin therapy was introduced to control hyperglycemia and ensure the survival of severely diabetic NOD mice with BG >30 mmol/L (41, 42), similar to the necessity of insulin therapy in human patients with T1D. The “Sliding Scale” protocol for insulin administration was necessary to keep the hyperglycemic animals alive throughout the experiment in all groups and to observe the effects of treatments during the long-term administration of the therapeutic agents and placebo. Importantly, the insulin therapy was only administered to mice in severe hyperglycaemia with BG levels over 30 mmol/L.

In the reversal arm of our study, we found that mice treated with the triple drug combination of GABA+DPP-4i+PPI received the least amount of exogenous insulin compared to all other dual drug combinations or placebo but had significantly higher circulating levels of C-peptide and endogenous insulin.

T1D clinical parameters and biomarkers were found to accurately correlate with T1D pancreas pathology (44). C-peptide, the molecule produced in an equimolar concentration to insulin, has become an established insulin secretion biomarker. Circulating levels of C-peptide in diabetic patients are widely used to determine the effects of interventions designed to preserve or improve residual β-cell function (45, 46), and the C-peptide response is often evaluated as the primary outcome in T1D intervention trials (10, 47, 48). Our findings, in the present study, of significant and superior actions of the triple drug combination of GABA, a DPP-4i and a PPI (A+B+C) to increase C-peptide and insulin levels, compared with the double drug combinations, both in diabetes prevention and reversal, supports the conclusion that the triple therapy improved diabetes by actions leading to improved islet β-cell function. Similar to other T1D murine studies investigating single agents and combination therapies (32, 33, 35), our experiment was conducted on non-stimulated β-cells in diabetic mice, and glucose tolerance testing was not included in the scope of our research. Immunohistochemical studies of pancreatic islet β-cell mass will be needed to evaluate possible regeneration and preservation of β cells after treatment with these different drug combinations.

T1D is a disease with numerous distinct pathogenic pathways underlying its clinical manifestation. We believe that the prevention of diabetes and its reversal may be explained by reports that the three therapeutic agents used in this study target different processes that occur in T1D.

The first component of the A+B+C combination, GABA, functions as an important transmitter within the islets, acting to regulate islet cell function. It promotes regeneration of β-cells, counters their apoptosis induced by cytokines, drugs, and other stressors, and has anti-inflammatory and immunoregulatory activities (49, 50). Furthermore, studies have indicated that GABA induces α-cell to β-cell transdifferentiation, beginning with the conversion of pancreatic duct cells to α-cells (32).

The second component of the A+B+C combination, sitagliptin, inhibits DPP-4, an enzyme strongly expressed on pancreatic islet cells and on the surface of immune cells (51). DPP-4 inhibition exerts positive effects on insulin secretion, β-cell survival and proliferation (52). Like other DPP-4i, sitagliptin prevents the degradation of the incretin hormone GLP-1 and thus prolongs stimulation of GLP-1 receptors (GLP-1R) with subsequent sustained elevation of cAMP and activation of the PI3K/AKT signaling pathway, which together activate key transcription factors for cell growth and survival (17). GLP-1 lowers blood glucose levels by stimulating insulin secretion from pancreatic β-cells in a glucose-dependent manner, while simultaneously inhibiting glucagon secretion from α-cells (53).

In addition, DPP-4i have been hypothesized to treat autoimmune T1D because they down-regulate Th1 cells, up-regulate Th2 cells and cytokines and promote Treg proliferation (19). Studies show that inhibition of CD26/DPP4 leads to decreased T-cell activation, proliferation, and migration, as well as increased GLP-1-mediated uptake of GABA by immune and endocrine cells (54, 55). We chose a dose of 30 mg/kg for the DPP-4i, sitagliptin, because this was the dose used by He and colleagues (56) to significantly downregulate serum IL-1β and IL-12 in NOD mice compared to placebo control.

Previously, the combination of GABA and sitagliptin was shown to promote regeneration of β-cell and reduce their apoptosis in the mouse model of streptozotocin (STZ)-induced β-cell injury (33) and in human islets transplanted into immunodeficient mice with STZ-induced diabetes (57). Subsequently, it was reported that these results are due, in part, to an additive effects of the agents to activate the PI3K/AKT pathway, stimulate cAMP-β-catenin signaling, reduce TxNIP activity, and promote SIRT1 and α-Klotho expression (55, 58). In the diabetes reversal arm of the current study, we observed that the combination of GABA and sitagliptin reduced blood glucose levels and lowered exogenous insulin demand, however the GABA and sitagliptin combination was significantly less effective than the triple combination therapy and did not significantly increase C-peptide and insulin levels in the mice.

We attribute the limited effects of the GABA and sitagliptin combination in our study, when compared to that of other studies (33, 57) to important differences between chemically-induced diabetes in the mice in those studies, and spontaneous autoimmune diabetes in the NOD mouse model of human T1D used in our study. Diabetes development in the NOD mouse provides insights into the functions of immune cells, showing many similarities to human autoimmune T1D. Chemically induced diabetic mice undergo initial β-cell destruction, but there is no autoimmune attack against the islet β-cells, so that β-cells do not suffer from a sustained and continued immune attack that would neutralize possible β-cell regeneration. Therefore, the therapeutic benefit of the GABA and sitagliptin combination in chemically-induced diabetic mice will not be reflective of the β-cell regeneration process in NOD mice and human T1D, where autoimmunity has to be countered to allow for possible β-cell regeneration.

The third component of the A+B+C combination, the PPI, omeprazole increases endogenous gastrin levels, which stimulates β-cell regeneration and improves glucose tolerance (59). Here, we investigated the addition of a PPI in form of omeprazole because it was previously reported to improve glycemic control by increasing gastrin levels in the blood and pancreas and has been shown to act as an adjuvant with hypoglycemic drugs (60–62). Additionally, omeprazole was reported to simultaneously increase insulin secretion by inhibiting the V-ATPase proton pump in β-cell insulin granules (63).

The combination of a DPP-4i with a PPI was examined before and found to restore normoglycemia in db/db diabetic mice (64) and in NOD mice (35). In agreement with the latter study in NOD mice (35), we found that the dual combination of a DPP-4i and a PPI in the present study was able to significantly reduce the blood glucose elevation in the prevention arm of the study in comparison with the placebo. However, the DPP-4i and PPI combination in the present study was not able to significantly decrease severe hyperglycemia in the diabetes reversal arm of the study, whereas the triple therapy of GABA, a DPP-4i and a PPI did significantly decrease blood glucose levels in both the diabetes reversal and prevention arms of the study. The different results reported in these two studies probably derive from a difference in experimental design. In the earlier study in NOD mice (35), a blood glucose level above 10 mmol/L was used to define diabetes onset and the start of therapies, whereas NOD mice in our study were defined to be diabetic only when blood glucose levels exceeded 13.9 mmol/L. Moreover, the test therapies in our study started when the entire reversal arm cohort of mice became diabetic with a median blood glucose level of 27.4 mmol/L for the A+B+C group and 28.0 mmol/L for the sitagliptin and omeprazole group, which represent severe hyperglycemia. Therefore, it is apparent that the A+B+C triple combination was needed to successfully treat severe hyperglycemia, whereas a dual drug combination of sitagliptin and omeprazole would suffice when starting from a much lower level of hyperglycemia (35).

## Conclusion

Our findings demonstrate superior efficacy of the combination of GABA, sitagliptin and omeprazole in prevention and reversal of diabetes in NOD mice when compared to double therapies. Additional glucose tolerance tests are recommended to assess the full extent of the triple combination therapy to improve stimulated β-cell function. GABA, DPP-4 inhibitors such as sitagliptin, and PPIs such as omeprazole can be taken orally, which is a significant clinical benefit. Toxicology studies on both DPP-4i and PPI have been extensively investigated, and clinical studies on GABA are currently underway. The promising findings in the present study warrant conducting clinical trials to examine the safety and effectiveness of the triple combination of GABA, sitagliptin and omeprazole as a novel pharmacological treatment for patients with insulin-dependent T1D and prevention for high-risk groups.

## Data availability statement

The raw data supporting the conclusions of this article will be made available by the authors, without undue reservation.

## Ethics statement

The animal study was reviewed and approved by I. M. Sechenov First Moscow State Medical University.

## Author contributions

Conceptualization: DK, LK, and SL; Data curation: FL-R, DK, LK, VT, AR, and SL; Formal analysis: FL-R, DK, LK, VT, AR, and SL; Funding acquisition: DK, LK, SL and HS; Investigation: FL-R, DK, AN, LK, VT, AR, HS, and SL; Methodology: FL-R, DK, AN, LK, and VT; Project administration: DK, LK, SL, and HS; Resources: DK, LK, VT, HS, and SL; Supervision: FL-R, DK, LK, VT, AR, HS, and SL; Validation: FL-R, DK, AN, LK, VT, AR, HS, and SL; Writing—original draft: FL-R, DK, LK, VT, AR, HS, and SL; Writing—review and editing: FL-R, DK, LK, VT, AR, HS, and SL. All authors contributed to the article and approved the submitted version.

## Funding

This study received funding from Levicure LTD (Israel). HS is supported by the Swedish Research Council and the Novo Nordisk Foundation. The funders were not involved in the study design, collection, analysis, interpretation of data, the writing of this article or the decision to submit it for publication. All authors declare no other competing interests.

## Acknowledgments

We thank Drs. Michael B. Zemel and Georgina Xanthou for their scientific guidance and review of this manuscript.

## Conflict of interest

Authors SL, DK, and LK are members of Levicure LTD and have patents related to the triple combination GABA, PPI, DPP4-i. VT was employed by Advanced Molecular Technology LLC.

The remaining authors declare that the research was conducted in the absence of any commercial or financial relationships that could be construed as a potential conflict of interest.

## Publisher’s note

All claims expressed in this article are solely those of the authors and do not necessarily represent those of their affiliated organizations, or those of the publisher, the editors and the reviewers. Any product that may be evaluated in this article, or claim that may be made by its manufacturer, is not guaranteed or endorsed by the publisher.
